# Polyglutamine toxicity assays highlight the advantages of mScarlet for imaging in *Saccharomyces cerevisiae*

**DOI:** 10.12688/f1000research.15829.2

**Published:** 2018-11-26

**Authors:** Maram B. Albakri, Yuwei Jiang, Julie Genereaux, Patrick Lajoie

**Affiliations:** 1 Department of Anatomy and Cell Biology, The University of Western Ontario, London, Ontario, N6A5C1, Canada; 2 Department of Biochemistry, The University of Western Ontario, London, Ontario, N6A5C1, Canada

**Keywords:** fluorescent proteins, mScarlet, yeast, polyglutamine toxicity, aggregation, Huntington’s disease

## Abstract

Development of fluorescent proteins (FPs) enabled researchers to visualize protein localization and trafficking in living cells and organisms. The extended palette of available FPs allows simultaneous detection of multiple fluorescent fusion proteins. Importantly, FPs are originally derived from different organisms from jelly fish to corals and each FP displays its own biophysical properties. Among these properties, the tendency of FPs to oligomerize inherently affects the behavior of its fusion partner. Here we employed the budding yeast *Saccharomyces cerevisiae* to determine the impact of the latest generation of red FPs on their binding partner. We used a yeast assay based on the aggregation and toxicity of misfolded polyQ expansion proteins linked to Huntington’s disease. Since polyQ aggregation and toxicity are highly dependent on the sequences flanking the polyQ region, polyQ expansions provide an ideal tool to assess the impact of FPs on their fusion partners. We found that unlike what is observed for green FP variants, yemRFP and yFusionRed-tagged polyQ expansions  show reduced toxicity.  However, polyQ expansions tagged with the bright synthetically engineered ymScarlet displayed severe polyQ toxicity. Our data indicate that ymScarlet might have significant advantages over the previous generation of red FPs for use in fluorescent fusions in yeast.

## Introduction

Following the development of the green fluorescent protein (GFP) from the jellyfish *Aqueaora victoria* ( Chalfie *et al*., 1994), several other FPs with various spectral properties have been characterized ( Thorn, 2017), allowing simultaneous detection of multiple fluorescent reporters. Among the most popular alternatives to GFP are the red fluorescent proteins (RFPs) isolated from *Anthozoa* coral and anemone species. One of the drawbacks of RFPs is that *Anthozoa* derived FPs are obligate tetramers ( Baird *et al*., 2000; Verkhusha & Lukyanov, 2004). While development of RFPs into monomeric versions has been successful, it is often associated with reduced brightness of the fluorescent signal ( Campbell *et al*., 2002) and therefore reduced overall performance of the resulting monomeric FPs. Moreover, RFPs such as TagRFP and mRuby2 reported as monomeric by passing purified proteins through sizing columns still display high tendency to oligomerize in living mammalian cells ( Costantini *et al*., 2015; Costantini *et al*., 2012). Thus, under specific circumstances, FPs reported as monomeric can still be prone to oligomerization. Unwanted formation of oligomers could potentially significantly alter the function/localization of the protein of interest fused to the FP and render reporters unreliable ( Costantini *et al*., 2015; Snapp *et al*., 2003; Zacharias *et al*., 2002). Indeed, various RFPs (mCherry, mKate2, mRuby, mKO2, mApple, TagRFP-T) have been shown to have differential effects on localization of cdc12 in yeast ( Lee *et al*., 2013). Thus, being able to assess the behavior of fluorescent reporter in a given organism and/or cellular compartment is critical to help optimize fluorescent reporter design ( Snapp, 2009).

We recently established a method to rapidly compare the behavior of FPs against a monomeric variant of superfolder GFP (msfGFP) in yeast ( Jiang *et al*., 2017). The assays exploit the ability of polyglutamine expansions associated with Huntington’s disease (HD) to form toxic aggregates in yeast cells. The cause of HD can be traced back to abnormal expansion of a polyQ stretch within the first exon of the gene encoding the Huntingtin protein (Htt ^ex1^) resulting in chorea and cognitive defects in patients ( Gusella & MacDonald, 1995; Huntington, 1872; Penney *et al*., 1997). Expansion over 36 repeats is known to cause the Htt protein to misfold and aberrantly accumulate into detergent-insoluble amyloid-like aggregates in the cytoplasm of striatal neurons ( Penney *et al*., 1997). Expression of expanded Htt ^ex1^ in yeast results in severe polyQ aggregation and growth defect ( Duennwald, 2013; Krobitsch & Lindquist, 2000; Mason & Giorgini, 2011; Meriin *et al*., 2002). Interestingly, the nature of the sequences flanking the polyQ regions (in this case fluorescent or epitope tags) greatly affects the propensity of the polyQ expansions to aggregate and to display significant growth defects in yeast ( Duennwald *et al*., 2006). Using polyQ toxicity assays in yeast, we previously showed that a yeast-optimized version of mCherry (termed yemRFP ( Keppler-Ross *et al*., 2008)) displays only a mild growth defects compared to yeast-optimized msfGFP (ymsfGFP) ( Jiang *et al*., 2017). These results lead us to exploit the polyQ toxicity and aggregation assays to explore the effects of two of the most recently available RFPs. Here, we focused on FusionRed, a red monomeric fluorescent variant of mKate2 known for its low cytotoxicity in cells ( Shemiakina *et al*., 2012) that displays low propensity to oligomerize in mammalian cells ( Costantini *et al*., 2015). We also included mScarlet, a monomeric synthetic RFP that was recently shown to outperform other RFPs in terms of brightness of the fluorescent signal ( Bindels *et al*., 2017). Both have yet to be characterized for expression in yeast.

## Methods

### Yeast strains and culture conditions

All strains are derived from W303-1A ( Thomas & Rothstein, 1989). All experiments were conducted in synthetic complete media (SC) at 30°C.

### DNA constructs

yemRFP ( Keppler-Ross *et al*., 2008) was previously described. yFusionRed and ymScarlet were codon optimized for expression in yeast and synthetized by Genscript Inc. based on previously published sequences ( Bindels *et al*., 2017; Shemiakina *et al*., 2012). RFPs were cloned into the SpeI/SalI site of p415 GPD. Alternatively, RFPs were cloned into the SpeI/SalI sites of p415 GAL1 25Q/68Q Htt ^ex1^ lacking the proline rich domain, as previously described ( Jiang *et al*., 2017). To generate 2µ vectors, the GAL1 25Q/68Q Htt ^ex1^-ymsfGFP or GAL1 25Q/68Q Htt ^ex1^-yFusionRed fragments were cloned into the SacI/SalI sites of pRS42N ( Taxis & Knop, 2006). All Htt ^ex1^ constructs lack the proline-rich domain since absence of this domain is required for Htt ^ex1^ toxicity in yeast ( Duennwald *et al*., 2006). We also noted that since the publication of our previous study ( Jiang *et al*., 2017), the original 72Q Htt ^ex1^ plasmid has mutated into 68Q. We, therefore, used the latter in this study. See Table 1 for a list of plasmids used in this study.

**Table 1.** Plasmids used in this study.

Plasmids	Resistance marker	Source
P415 GPD	Leu	( Mumberg *et al.*, 1995)
P415 GPD-yemRFP		This study
P415 GPD-yFusionRed		
P415 GPD-ymScarlet		
P415 Gal1-FLAG-25Q-ymsfGFP		( Jiang *et al.*, 2017)
P415 Gal1-FLAG-68Q-ymsfGFP		This study
P415 Gal1-FLAG-25Q-yemRFP		( Jiang *et al.*, 2017)
P415 Gal1-FLAG-68Q-yemRFP		This study
P415 Gal1-FLAG-25Q-yFusionRed		
P415 Gal1-FLAG-68Q-yFusionRed		
P415 Gal1-FLAG-25Q-ymScarlet		
P415 Gal1-FLAG-68Q-ymScarlet		
PRS42N Gal1-FLAG-25Q-ymsfGFP	natNT2	
PRS42N Gal1-FLAG-68Q-ymsfGFP		
PRS42N Gal1-FLAG-25Q-yFusionRed		
PRS42N Gal1-FLAG-68Q-yFusionRed		

### Growth assays

Yeast growth was measured by spotting assay on agar plates. Briefly, cells were cultured overnight to saturation in appropriate selection media. The next day, cells densities were equalized to OD _600_ 0.2 and 5x serial dilutions were spotted on agar plates. Alternatively, cell densities were equalized to OD _600_ 0.1 and cells 300 µL of cell suspensions were transferred into a 96 well plate and incubated at 30 ^o^C for 24h with constant shaking in a Biotek Epoch 2 microplate spectrophotometer and OD _600_ was recorded every 15 minutes.

### Dot blot

After induction in galactose media overnight, cells were lysed using glass beads in lysis buffer (100 mM Tris pH 7.5; 200 mM NaCl; 1 mM EDTA; 5% glycerol, 1 mM Dithiothreitol (DTT) 4 mM phenylmethylsulfonyl fluoride (PMSF) and protease inhibitor cocktail). Equal amount of proteins were spotted on a nitrocellulose membrane. Membranes were blocked for 30 min in PBS-0.05%Tween at room temperature were and then processed for immunoblot. Membranes were probed with anti-FLAG primary antibody (Sigma F3040, 1:5000 dilution) overnight at 4°C and subsequently with a secondary anti-mouse fluorescent antibody (Thermo Alexa 555 #A21424, 1:5000 dilution) for 1h at room temperature and imaged using a Bio-Rad ChemiDoc MP imaging system. Membranes were then stripped using the Gene Bio-Application stripping buffer and reprobed with an anti-Pgk1 primary antibody (Thermo 22C5D8) using the same secondary antibody. In Figure 3, for each individual antibody, both membranes were imaged simultaneously to allow direct comparison of fluorescent signal. Densitometric analysis was performed using Image J.

### Fluorescence microscopy

**Figure 1.**
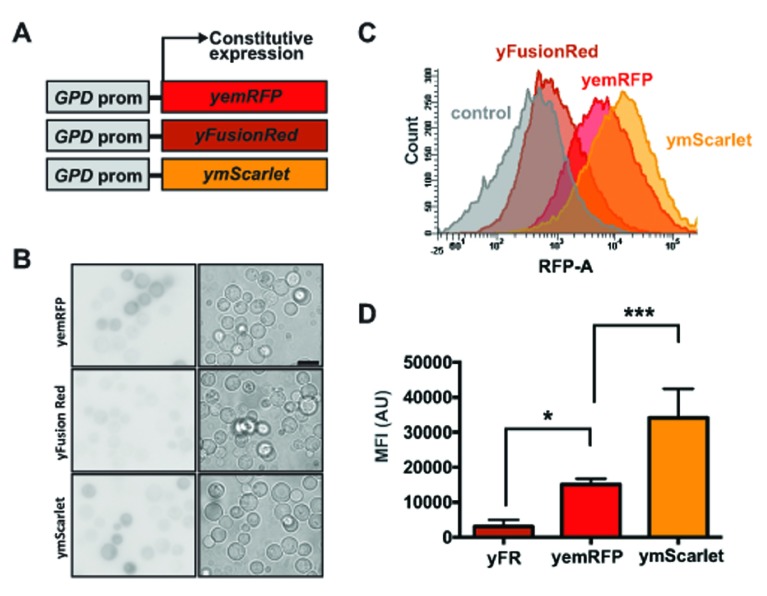
Comparison of red fluorescent proteins (RFPs) fluorescent intensities in yeast. ( **A**) Scheme of fluorescent proteins vectors. yemRFP, yFusionRed and ymScarlet were introduced into centromeric vectors under the control of a constitutive GPD promoter. ( **B**) Representative images from 3 fields of yeast cells expressing different RFPs. Imaging conditions were kept constant between samples to allow direct comparison of fluorescent intensities. Inverted black and white images are shown for clarity. Bar: 5µm ( **C**) Yeast cells expressing the different RFPs were analyzed by flow cytometry and compared to cells carrying an empty vector. ( **D**) Median fluorescent intensities of the various RFPs were calculated from fluorescent data acquired using flow cytometry. *p<0.05 and ***p<0.001 according to a one way ANOVA followed by a Dunnett’s multiple comparison test comparing samples to yFusionRed.

**Figure 2.**
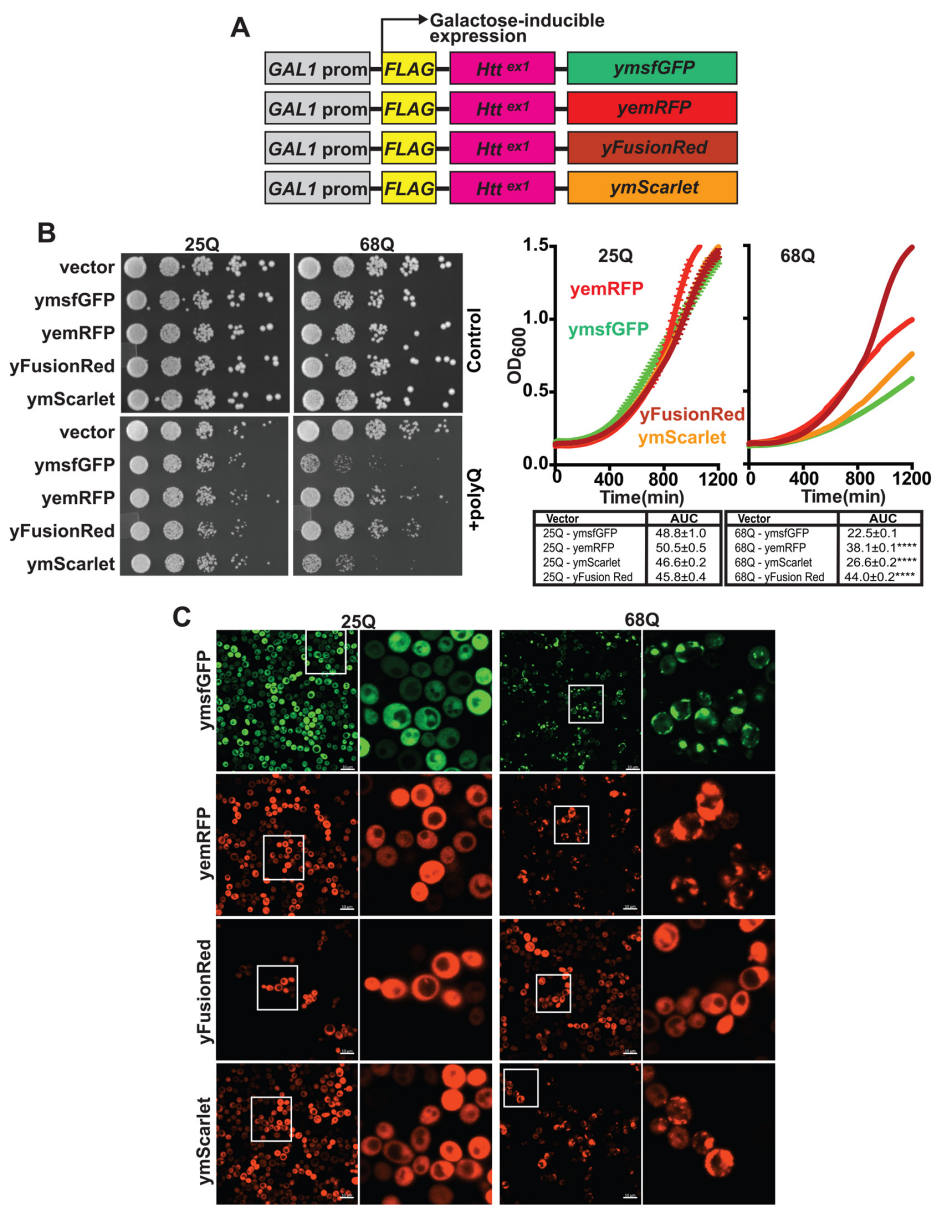
Unlike yFusionRed, ymScarlet displays a toxic polyQ phenotype similar to ymsfGFP. ( **A**) Scheme of vectors encoding fluorescent Fluorescent proteins (FPs) were cloned in frame with FLAG-Htt ^ex1^ into a centromeric vector carrying a *GAL1* inducible promoter. ( **B**) Images of yeast growth assays on agar plates. Yeast cells carrying an empty vector or 25/68Q Htt ^ex1^ fused to either ymsfGFP, yemRFP, yFusionRed or ymScarlet were grown to saturation overnight in glucose (control) or galactose (polyQ induced) containing media. The next days, cell concentrations were equalized to OD _600_ 0.2 and 5 fold serial dilutions of the cell suspension spotted on synthetic complete agar media plates containing either glucose or galactose. Alternatively, cells were cultured in liquid media and optical densities were recorded over time to generate growth curves. The area under the curve (AUC) was calculated from 3 experimental replicates. ****p<0.0001 according to a one way ANOVA followed by a Dunnett’s multiple comparison test comparing samples to ymsfGFP-tagged fusion of the same polyQ length. ( **C**) Representative fluorescent images from 3 fields of yeast cells expressing 25/68Q Htt Htt ^ex1^ fused to ymsfGFP, yemRFP, yFusionRed or ymScarlet after overnight induction in galactose-containing media.

Under the different experimental conditions, cells were diluted 10x in growth media and plated in Lab-tek (Thermo Inc.) imaging chambers and processed for fluorescence microscopy. Images in Figure 1 were acquired using a Zeiss AxioVert A1 wide field fluorescence microscope equipped with a 63X NA 1.4 Plan Apopchromat objective, a 560 to 600nM excitation/630 to 705 nm emission bandpass filter and Zeiss Axiocam 506 mono camera. Images presented in Figure 2 and Figure 4 were collected using a Zeiss 800 confocal microscope equipped with 488 nm and 561 nm diode lasers and a 63x PlanApochromat NA 1.4 objective.

**Figure 3.**
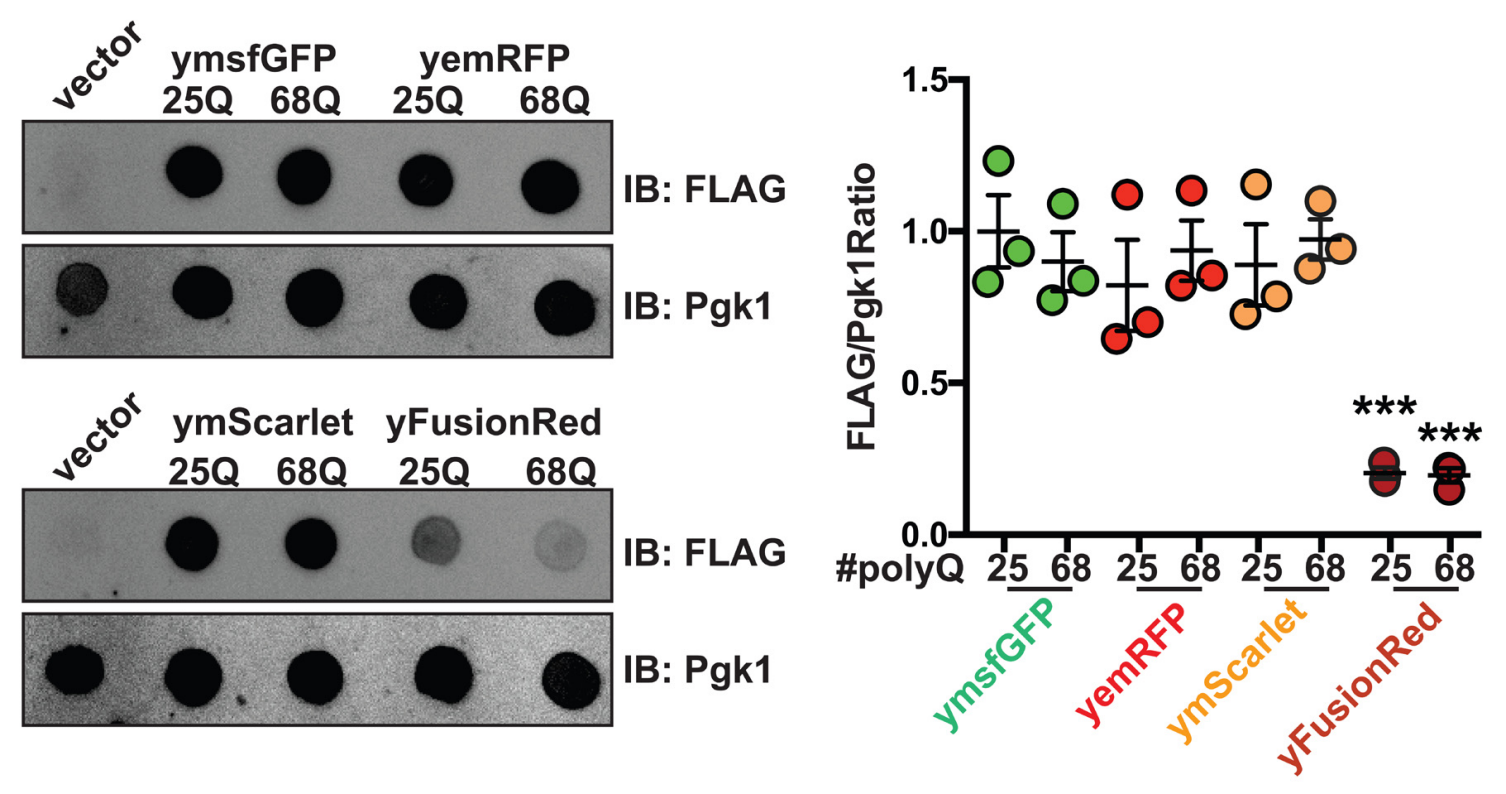
yFusionRed-tagged Htt ^ex1^ fusions are expressed at lower levels compared to other fluorescent fusions. ( **A**) Yeast cells carrying an empty vector or 25/68Q Htt ^ex1^ fused to ymsfGFP, yemRFP, yFusionRed or ymScarlet or carrying an empty vector were induced overnight in galactose containing media and protein levels analyzed by dot blot using either an anti-FLAG (detection of fluorescent fusions) or anti-Pgk1 antibody (loading control). Quantification of the FLAG/Pgk1 ration is shown from 3 independent experiments. ***p<0.001 according to a one way ANOVA followed by a Tukey’s multiple comparison test comparing samples to ymsfGFP-tagged fusion of the same polyQ length.

**Figure 4.**
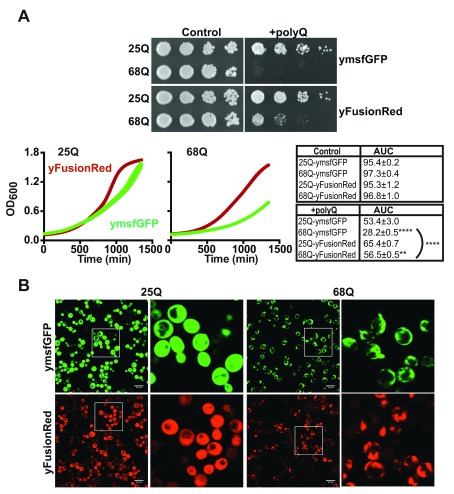
Expanded Htt ^ex1^-yFusionRed is toxic when expressed at high levels. ( **A**) Images of yeast growth assays on agar plates. Yeast cells carrying 25/68Q Htt ^ex1^ fused to either ymsfGFP or yFusionRed in a 2µ multicopy vector were grown to saturation overnight in glucose (control). The next days, cell concentrations were equalized to OD _600_ 0.2 and 5 fold serial dilutions of the cell suspension spotted on synthetic complete agar media plates containing either glucose (control) or galactose (+polyQ). Alternatively, cells were cultured in liquid media and optical densities were recorded over time to generate growth curves. The area under the curve (AUC) was calculated from 3 experimental replicates. **p<0.01 and ****p<0.0001 according to a one way ANOVA followed by a Tuckey’s multiple comparison test comparing the 68Q sample to its 25Q counterpart unless indicated otherwise. ( **B**) Representative fluorescent images from 3 fields of yeast cells expressing 25/68Q Htt Htt ^ex1^ fused to ymsfGFP or yFusionRed from a multicopy 2µ vector after overnight induction in galactose-containing media. Under these conditions, 68Q-yFusionRed displays robust aggregation.

### Flow cytometry

Cell were cultured with appropriate media and processed for flow cytometry using a BD Bioscience FACS Celesta flow cytometer equipped with a 561 Yellow laser for imaging of RFPs. Data were analyzed using the BD FACS Diva software. All conditions were performed in triplicates, 20,000 cells were analyzed and median fluorescence intensities were calculated. No gates were applied.

### Statistical analysis

A one way ANOVA followed by a multiple comparison test (Tukey’s or Dunnett’s according to figure legends) was used to determine statistical significance between the different experimental conditions in Figure 1D, Figure 2B, Figure 3 and Figure 4A using GraphPad Prism v6.0h.

## Results and discussion

To analyze the performance of the three different RFPs in yeast, we first generated codon optimized versions of both FusionRed and mScarlet (termed yFusionRed and ymScarlet, respectively) ( Table 2). Centromeric plasmids encoding yFusionRed, yemRFP and ymScarlet under the control of the constitutive GPD promoter were transformed in yeast ( Figure 1A). Fluorescence intensities were compared using wide-field fluorescence microscopy ( Figure 1B). Median fluorescence intensity (MFI) was then quantified using flow cytometry. Quantification revealed that yFusionRed was significantly dimmer (~5x) than yemRFP ( Figure 1C and D). This result was surprising given that previously published data reported a slightly increased brightness for FusionRed when compared to mCherry ( Shemiakina *et al*., 2012). However, it is known that fluorescent brightness of FPs expressed in yeast can be different from the ones registered for pure purified proteins ( Lee *et al*., 2013). As opposed to yFusionRed, ymScarlet displayed the strongest fluorescent signal (~2x brighter than yemRFP) ( Figure 1C and D). These results are in agreement with previous studies reporting increased brightness of mScarlet compared to other RFPs variants ( Bindels *et al*., 2017). Based on the intensity of the fluorescent signal, ymScarlet appears to be the optimal RFP for imaging in yeast.

**Table 2.** Sequences of yeast optimized fluorescent proteins generated in this study.

Name	Sequences
yFusionRed	ATGGTTTCTGAATTGATTAAAGAAAACATGCCAATGAAGTTGTACATGGAAGGTACTGTTAACAACCATCATTTTAAATGTACATC AGAAGGTGAAGGTAAACCATACGAAGGTACTCAAACAATGAGAATTAAAGTTGTTGAAGGTGGTCCATTGCCATTTGCTTTCGA TATTTTGGCAACTTCTTTTATGTACGGTTCAAGAACTTTTATTAAGCATCCACCAGGTATTCCAGATTTCTTTAAGCAATCTTTCCCA GAAGGTTTTACTTGGGAAAGAGTTACTACATATGAAGATGGTGGTGTTTTGACTGCAACACAAGATACATCATTGCAAGATGGTT GTTTGATCTATAACGTTAAAGTTAGAGGTGTTAATTTTCCAGCTAATGGTCCAGTTATGCAAAAGAAAACTTTGGGTTGGGAAGC TTCTACTGAAACAATGTACCCAGCAGATGGTGGTTTAGAAGGTGCTTGTGATATGGCATTGAAATTGGTTGGTGGTGGTCATTTG ATCTGTAATTTGGAAACTACATACAGATCTAAGAAACCAGCTACAAATTTGAAGATGCCAGGTGTTTACAACGTTGATCATAGATT GGAAAGAATTAAAGAAGCAGATGATGAAACTTACGTTGAACAACATGAAGTTGCTGTTGCAAGATACTCTACAGGTGGTGCTG GTGACGGTGGTAAATAA
ymScarlet	ATGGTTTCTAAAGGTGAAGCAGTTATTAAGGAATTCATGAGATTCAAGGTACACATGGAAGGTTCTATGAATGGTCACGAATTTG AAATTGAAGGTGAAGGTGAAGGTAGACCATATGAAGGTACTCAAACTGCTAAGTTGAAGGTTACTAAAGGTGGTCCATTGCCAT TTTCTTGGGATATTTTGTCTCCACAATTCATGTACGGTTCTAGAGCTTTTACAAAACATCCAGCAGATATTCCAGATTACTACAAGC AATCATTCCCAGAAGGTTTTAAATGGGAAAGAGTTATGAACTTCGAAGATGGTGGTGCAGTTACTGTTACACAAGATACTTCTTT GGAAGATGGTACATTGATCTATAAGGTTAAGTTGAGAGGTACTAATTTTCCACCAGATGGTCCAGTTATGCAAAAGAAAACTATG GGTTGGGAAGCTTCAACAGAAAGATTGTACCCAGAAGATGGTGTTTTGAAGGGTGACATTAAGATGGCATTGAGATTGAAGGA TGGTGGTAGATATTTGGCTGATTTCAAGACTACATACAAGGCTAAGAAACCAGTTCAAATGCCAGGTGCTTACAACGTTGATAGA AAGTTGGATATTACTTCTCATAATGAAGATTACACAGTTGTTGAACAATATGAAAGAAGTGAAGGTAGACACAGTACAGGTGGTAT GGATGAATTATACAAATGA

Raw data behind Figures 1, 2, 3 and 4 Click here for additional data file. Copyright: © 2018 Albakri MB et al. 2018 Data associated with the article are available under the terms of the Creative Commons Zero "No rights reserved" data waiver (CC0 1.0 Public domain dedication).

Next, we sought to determine how the three different FPs affect their fusion partners in living yeast. To this end, we employed the polyQ toxicity assays. Each RFP was cloned in frame with a galactose inducible version of Htt ^ex1^ carrying either 25Q (non-pathological length) or 68Q (HD-associated) ( Figure 2A). 25Q constructs show no growth differences across the different FPs in both uninduced (glucose media) and polyQ-induced (galactose media) conditions indicating that expression of the different constructs results in similar growth phenotypes. When fused to 68Q Htt ^ex1^, yFusionRed displayed no significant toxicity when compared to the non-toxic 25Q fusion ( Figure 2B). Interestingly, ymScarlet displayed severe toxicity, showing a slow growth phenotype comparable to what was observed for ymsfGFP ( Figure 2B). Based on these observations, we then investigated the effects of the different RFPs on polyQ aggregation using fluorescence microscopy. We found that yemRFP displayed robust 68Q aggregation similar to ymsfGFP as we previously described ( Jiang *et al*., 2017). It is important to note that while prone to aggregation, yemRFP polyQ proteins were shown to form aggregates with different biophysical properties (increased detergent solubility) that can account for their moderately toxic nature ( Jiang *et al*., 2017). In accordance with the absence of toxicity noted in the growth assay, 68Q-FusionRed did not form visible aggregates, while ymScarlet displayed strong aggregation propensity ( Figure 2C). In addition, assessment of protein abundance for each constructs using dot blot revealed that both 25 and 68Q yFusionRed fusions were present at lower levels compared to other fluorescent counterparts ( Figure 3A). The cause of this phenotype is unclear and could result from increased turnover rate of the fusions. Interestingly, expression of 69Q-yFusionRed from a multicopy 2µ vector resulted in a growth defect, albeit toxicity was reduced compared to ymsfGFP-tagged polyQ ( Figure 4A). Moreover, under these conditions, 68Q-yFusionRed displayed robust aggregation. This indicates that the lower expression levels observed for yFusionRed constructs can potentially explain, at least partially, the absence of polyQ toxicity when expressed at lower levels. Reduced toxicity of the 68Q-yFusionRed is also consistent with our previous observation showing that yomTagBFP2, a blue fluorescent proteins similarly does not form toxic aggregates ( Jiang *et al*., 2017). In fact, both FusionRed and mTagBFP2 ( Subach *et al*., 2011) are evolved versions of the wild-type RFP from sea anemone *Entacmaea quadricolor* ( Merzlyak *et al*., 2007). In the case of mScarlet, the protein was evolved from a synthetic template design for generating a monomeric protein. Therefore, based on our data, mScarlet appears to be an attractive alternative to mCherry, which minimizes the effect of the FP on its fusion partner.

## Data availability

The data referenced by this article are under copyright with the following copyright statement: Copyright: © 2018 Albakri MB et al.

Data associated with the article are available under the terms of the Creative Commons Zero "No rights reserved" data waiver (CC0 1.0 Public domain dedication). 

F1000Research: Dataset 1. Raw data behind Figure 1, Figure 2, Figure 3 and Figure 4, https://doi.org/10.5256/f1000research.15829.d225385 ( Albakri *et al*., 2018)

Both p415 GPD-yFusionRed and p415 GPD-ymScarlet are available from addgene (#111916/11917). All plasmids are available upon request from the corresponding author.
