# Supplementary material for: Polyglutamine toxicity assays highlight the advantages of mScarlet for imaging in Saccharomyces cerevisiae
Source: F1000Res. 2018 Nov 26;7:1242. Originally published 2018 Aug 10. [Version 2] doi: 10.12688/f1000research.15829.2 (PMC6290977; doi:10.12688/f1000research.15829.2)
Supplement: Peer review history

# Peer review: Referee response for version 2

**DOI:** 10.5256/f1000research.17889.r41113

**Published:** 2018-12-07

**Authors:** Danny M. Hatters

**Affiliations:** 1 Dept of Biochem & Mol Biology, The University of Melbourne, Melbourne, VIC, 3010, Australia

I have read the revised version and I am happy with the changes the authors have made and I now recommend the work be indexed.

I have read this submission. I believe that I have an appropriate level of expertise to confirm that it is of an acceptable scientific standard.

# Peer review: Referee response for version 2

**DOI:** 10.5256/f1000research.17889.r41114

**Published:** 2018-11-27

**Authors:** Fedor V. Subach

**Affiliations:** 1 Moscow Institute of Physics and Technology (MIPT), Moscow, Russian Federation

I have no further comments to make.

I have read this submission. I believe that I have an appropriate level of expertise to confirm that it is of an acceptable scientific standard.

# Peer review: Referee response for version 1

**DOI:** 10.5256/f1000research.17277.r37082

**Published:** 2018-08-21

**Authors:** Fedor V. Subach

**Affiliations:** 1 Moscow Institute of Physics and Technology (MIPT), Moscow, Russian Federation

In this manuscript Albakri Maram et al. demonstrated superior performance of the mScarlet red fluorescent protein over the FusionRed protein in the Huntington’s disease related yeast assay. First, authors showed the higher brightness of non-fused mScarlet protein vs FusionRed during constitutive expression in yeast. Albakri Maram et al. then made fusions of the mScarlet and FusionRed proteins with first exon of the Huntingtin protein (Httex1) carrying FLAG-tag and either non-pathological 25 repeats of glutamines or Huntington’s disease-associated 68 repeats of glutamines. Authors expressed these fusions in yeast and found that in opposite to the FusionRed protein, mScarlet displayed right cytotoxicity and polyQ aggregation. Overall, mScarlet red fluorescent protein can be used as a right tag in polyQ yeast assay for the Huntington’s disease studies. The strategy suggested in this paper may be used researchers for characterization of the properties of other newly developed fluorescent markers.

 Major points: 

 1) Results and discussion section, page 6, right column. Analyzing protein expression level on dot blot authors revealed lower levels of yFusionRed protein as compared to other RFPs and based on this fact they suggested its increased degradation in yeast cells (actually, one cannot exclude that yFusionRed lower expression level is not related with its high degradation on RNA level not protein level). These findings suggest that lower expression level of yFusionRed protein in yeast my prevent FusionRed protein from toxicity and formation of aggregates, which could be observed at higher FusionRed protein concentrations only. So we cannot say unambiguously about the real reasons of the absence of toxicity and aggregates formation in the case of FLAG-68Q-yFusionRed fusion.

 Lower expression level of yFusionRed also may masks its tendency to oligomerize inherently and prevent from correct comparison in this respect with other fluorescent proteins.

 Minor points: 

 1) Introduction section, page 3, left column, last sentence, please, replace misprint “…and aggregation assays to explore to effects of…” with “…and aggregation assays to explore the effects of…”.

 2) Methods section, page 3, right column, Yeast strains and culture conditions section, authors use term W303A. However according to provided reference it should be named as W303-1A. Please, correct.

 3) Methods section, page 3, right column, DNA constructs section, authors mention that they do not use proline rich domain. Please, add explanation of the reason of this.

 4) Methods section, page 3, right column, Dot blot section, please, replace misprint “…PSMF…” with “…PMSF…”.

 5) Methods section, page 3, right column, Dot blot section, please, replace misprint “…at room temperature were then processed…” with “…at room temperature and then processed …”.

 6) Methods section, page 3, right column, Fluorescent microscopy section and elsewhere in the text, please, replace misprint “…Fluorescent microscopy…” with “…Fluorescence microscopy…”.

 7) Methods section, page 4, Table 1. I would recommend to change the abbreviations of the constructs in order to mention Httex1 and avoid 25Q abbreviation which can be mistakenly interpreted as a substitution of the residue in position 25 with Q, i.e. for example, replace “p415 Gal1-FLAG-25Q-ymsfGFP” with “p415 Gal1-FLAG-Httex1-(Q)25-ymsfGFP”. 8) Methods section, page 5, Figure 2, panel A. I would recommend replace “Httex1” with “Httex1(Q)25/(Q)68”. 9) Methods section, page 5, Figure 2, panel A and elsewhere in the text. In accordance to the 6th point I would recommend replace “25Q” and “Q68” with “(Q)25” and “(Q)68”.

 10) Methods section, page 5, Legend to the Figure 1. Please, add explanation that panel (A) represents a “Scheme of fluorescent proteins expression vectors …”. Also, please, be consistent in the same respect for the panel (C).

 11) Methods section, page 5, legend to the Figure 2. Please, add explanation that panel (A) represents a “Scheme of fluorescent proteins expression system …”, panel (B) – “Image of yeast colonies …” and panel (C) – “Dot blots of …”.

 12) Results and discussion section, page 6, left column and elsewhere in the text and figure legends, please, replace misprint “Fluorescent intensities…” with “…Fluorescence intensities…”. 

 13) Results and discussion section, page 6, left column, authors mention that “…yFusionRed was significantly dimmer than yemRFP”. Below they write about the strongest fluorescent signal for ymScarlet. Please, provide these comparisons in more quantitative and scientific manner, i.e. mention in how many fold yFusionRed and ymScarlet RFPs were dimmer and brighter than yemRFP, respectively. 

 14) Results and discussion section, page 6, left column, please, replace misprint “… in yeast can differed from …” with “… in yeast can be different from …”.

 15) Results and discussion section, page 6, left column, please, replace misprint “… of mScarlet compared to other RFs…” with “…of mScarlet compared to other RFs …”.

 16) Results and discussion section, page 6, right column. Authors use 68 repeats of Q however in their previous paper of the year 2017 they utilized 72 Q repeats. Please, include in the text explanation why did you choose 68 not 72 repeats. It is important for the comparison of the data.

 17) Results and discussion section, page 6, right column. Authors write that “…yFusionRed displayed no significant toxicity…”. The term “significant” suggests statistical analysis, could you, please, provide which statistic criterion was used in this case, e.g. a two-tailed student t-test

 or Mann-Whitney or someone else? Now Statistical analysis section describes Figure1D data only.

 Overall, if the points raised above will be adequately addressed, indexing in F1000Research is appropriate.

I have read this submission. I believe that I have an appropriate level of expertise to confirm that it is of an acceptable scientific standard.

# Response

**Published:** 2018-11-08

**Authors:** Patrick Lajoie

**Affiliations:** University of Western Ontario

***We thank the reviewer for helpful comments. We have addressed concerns in the new version of the manuscript. See below for the authors’ response to specific points raised during the review process.***

 Major points: 

 1) Results and discussion section, page 6, right column. Analyzing protein expression level on dot blot authors revealed lower levels of yFusionRed protein as compared to other RFPs and based on this fact they suggested its increased degradation in yeast cells (actually, one cannot exclude that yFusionRed lower expression level is not related with its high degradation on RNA level not protein level). These findings suggest that lower expression level of yFusionRed protein in yeast my prevent FusionRed protein from toxicity and formation of aggregates, which could be observed at higher FusionRed protein concentrations only. So we cannot say unambiguously about the real reasons of the absence of toxicity and aggregates formation in the case of FLAG-68Q-yFusionRed fusion. Lower expression level of yFusionRed also may masks its tendency to oligomerize inherently and prevent from correct comparison in this respect with other fluorescent proteins.

***We agree with the reviewer on this point. We have now performed additional experiments where polyQ fusions are expressed using multicopy plasmids (2µ) were cells can express up to 50 copies of the constructs. Interestingly, under these conditions, 72Q-yFusionred can indeed aggregate and induce toxicity, albeit to lower extant than ymsfGFP-tagged polyQ counterparts. Therefore it is reasonable to hypothesize that lower expression of yFusionRed (maybe due to increased protein turnover) is responsible for decreased toxicity. Further experiments beyond the scope of this research note will be required to explain the relative lower expression of FusionRed in yeast.***

 Minor points: 

 1) Introduction section, page 3, left column, last sentence, please, replace misprint “…and aggregation assays to explore to effects of…” with “…and aggregation assays to explore the effects of…”.

***We have modified accordingly.***

 2) Methods section, page 3, right column, Yeast strains and culture conditions section, authors use term W303A. However according to provided reference it should be named as W303-1A. Please, correct.

***We have modified accordingly.***

 3) Methods section, page 3, right column, DNA constructs section, authors mention that they do not use proline rich domain. Please, add explanation of the reason of this.

***We added the rationale for the use of the Δ proline constructs which is described in Duennwald et al., 2006 PNAS.***

 4) Methods section, page 3, right column, Dot blot section, please, replace misprint “…PSMF…” with “…PMSF…”.

***We have modified accordingly.***

 5) Methods section, page 3, right column, Dot blot section, please, replace misprint “…at room temperature were then processed…” with “…at room temperature and then processed …”.

***We have modified accordingly.***

 6) Methods section, page 3, right column, Fluorescent microscopy section and elsewhere in the text, please, replace misprint “…Fluorescent microscopy…” with “…Fluorescence microscopy…”.

***We have modified accordingly***.

 7) Methods section, page 4, Table 1. I would recommend to change the abbreviations of the constructs in order to mention Httex1 and avoid 25Q abbreviation which can be mistakenly interpreted as a substitution of the residue in position 25 with Q, i.e. for example, replace “p415 Gal1-FLAG-25Q-ymsfGFP” with “p415 Gal1-FLAG-Httex1-(Q)25-ymsfGFP”. 8) Methods section, page 5, Figure 2, panel A. I would recommend replace “Httex1” with “Httex1(Q)25/(Q)68”. 9) Methods section, page 5, Figure 2, panel A and elsewhere in the text. In accordance to the 6th point I would recommend replace “25Q” and “Q68” with “(Q)25” and “(Q)68”.

***We thank the reviewer for the suggestion but the authors believe that to be consistent with the literature in the field, the current nomenclature should be used.***

 10) Methods section, page 5, Legend to the Figure 1. Please, add explanation that panel (A) represents a “Scheme of fluorescent proteins expression vectors …”. Also, please, be consistent in the same respect for the panel (C).

***We have modified accordingly.***

 11) Methods section, page 5, legend to the Figure 2. Please, add explanation that panel (A) represents a “Scheme of fluorescent proteins expression system …”, panel (B) – “Image of yeast colonies …” and panel (C) – “Dot blots of …”.

 12) Results and discussion section, page 6, left column and elsewhere in the text and figure legends, please, replace misprint “Fluorescent intensities…” with “…Fluorescence intensities…”. 

***We have modified accordingly.***

 13) Results and discussion section, page 6, left column, authors mention that “…yFusionRed was significantly dimmer than yemRFP”. Below they write about the strongest fluorescent signal for ymScarlet. Please, provide these comparisons in more quantitative and scientific manner, i.e. mention in how many fold yFusionRed and ymScarlet RFPs were dimmer and brighter than yemRFP, respectively. 

***We added values in the text.***

 14) Results and discussion section, page 6, left column, please, replace misprint “… in yeast can differed from …” with “… in yeast can be different from …”.

***We have modified accordingly.***

 15) Results and discussion section, page 6, left column, please, replace misprint “… of mScarlet compared to other RFs…” with “…of mScarlet compared to other RFs …”.

***We have modified accordingly.***

 16) Results and discussion section, page 6, right column. Authors use 68 repeats of Q however in their previous paper of the year 2017 they utilized 72 Q repeats. Please, include in the text explanation why did you choose 68 not 72 repeats. It is important for the comparison of the data.

***The polyQ repeats are known to be unstable. Sequencing of the DNA revealed that our 72Q original plasmid has mutated into 68Q. This change does not impact the toxicity of the fusion. We added a note in the material and methods section.***

 17) Results and discussion section, page 6, right column. Authors write that “…yFusionRed displayed no significant toxicity…”. The term “significant” suggests statistical analysis, could you, please, provide which statistic criterion was used in this case, e.g. a two-tailed student t-test

 or Mann-Whitney or someone else? Now Statistical analysis section describes Figure1D data only.

***Statistical analysis was performed on the growth curve data and described in the updated figure legends.***

# Peer review: Referee response for version 1

**DOI:** 10.5256/f1000research.17277.r37079

**Published:** 2018-08-17

**Authors:** Danny M. Hatters, Dezerae Cox

**Affiliations:** 1 Dept of Biochem & Mol Biology, The University of Melbourne, Melbourne, VIC, 3010, Australia

This study reports the impact of three different red fluorescent protein (RFP) tags on the expression and toxicity patterns of Httex1 in yeast by benchmarking them against the green fluorescent protein tagged Httex1 as the established model system in the literature. The authors reveal some differences in their effects on Httex1 toxicity and these results are likely to be of interest to those in the Httex1-polyQ field.

 Overall, the manuscript is well-written and figures are clearly presented. It should be noted that the phenomena reported here is a direct extension of a broader study of other fluorescent protein fusions previously reported by the same group (and has been been referenced).

 Major queries:

1. The authors state that the median fluorescent intensities if Fig 1 “reveals yFusionRed was significantly dimmer than yemRFP …  ymScarlet displayed the strongest fluorescent signal”. As written, this implies the median fluorescence intensity provides a measure of the relative brightness of individual fluorophores. However, without quantification of the amount of protein expressed this difference in median fluorescence could simply reflect differences in total fluorophore abundance. We are not familiar with yeast transfection methods - but certainly in mammalian transfection experiments cells will invariably have a massive (many fold) variation in expression levels within populations and between different constructs including a notable population of cells that have no expression. If this is the case in yeast, then the interpretation of difference in brightness is not sufficiently supported by the data. This point needs clarification.
2. Following from the above point, it is difficult to ascertain whether the differences in expression, toxicity and aggregate extents of the various fluorescent protein tagged Htt constructs (Fig 2) arises from the same issues. To us this would be a more likely (and simpler) explanation than what the authors explain as the protein constructs having differences in inherent toxicity (molecule per molecule). Further clarification of this issue is required.   
3. The level of reproducibility should be indicated by including at least 3 independent replicates for the dot blots and growth assays (in Fig 2).  Also, the dot blots should be quantified with the appropriate statistical methods.
4.  In Fig 1D, the authors report a significant difference in the median fluorescent intensities using a Student’s t-test to compare only two of the RFPs examined. It is suggested that an ANOVA would be more appropriate because there are more than two groups of interest. Based on the data provided, our ANOVA reports a higher degree of statistical significance for the comparison currently reported (yemRFP v ymScarlet, **) and also supports a significant difference between ymFusionRed and ymScarlet (***).

 

 Minor suggestions:

1. This sentence in the abstract has an unclear meaning as it implies ymScarlet is inherently affected by polyQ: “We found that unlike yemRFP and yFusionRed, the synthetically engineered ymScarlet displayed severe polyQ toxicity and aggregation similar to what is observed for green FP variants.”  Thus the sentence should be revised.
2. Additional details for the Dot blot method (secondary antibody for Pkt-1, dilutions) would enhance reproducibility
3. Include dimension for scale bar in Figure 2D

We have read this submission. We believe that we have an appropriate level of expertise to confirm that it is of an acceptable scientific standard, however we have significant reservations, as outlined above.

# Response

**Published:** 2018-11-08

**Authors:** Patrick Lajoie

**Affiliations:** University of Western Ontario

***We would like to thank the reviewers for their helpful comments. Below is our point-by-point response addressing the concerns raised during the review process.***

 Major queries: 

1. The authors state that the median fluorescent intensities if Fig 1 “reveals yFusionRed was significantly dimmer than yemRFP …  ymScarlet displayed the strongest fluorescent signal”. As written, this implies the median fluorescence intensity provides a measure of the relative brightness of individual fluorophores. However, without quantification of the amount of protein expressed this difference in median fluorescence could simply reflect differences in total fluorophore abundance. We are not familiar with yeast transfection methods - but certainly in mammalian transfection experiments cells will invariably have a massive (many fold) variation in expression levels within populations and between different constructs including a notable population of cells that have no expression. If this is the case in yeast, then the interpretation of difference in brightness is not sufficiently supported by the data. This point needs clarification.

***Yeast transformations are similar to E. coli transformations that the reviewers must be familiar with. All cells in the population are expressing the contructs since the cells are kept under constant selection. Since all constructs are expressed under the same promoter in a low copy centromeric vector (1-2 copies per cell), there is no reason to believe that some reason yFusionRed cells do not express fusion at the mRNA level. Quantification of the relative protein levels is problematic since anti-RFP antibodies could recognize mScarlet and yFusionred with different affinities. Addition of an additional epitope tag could affect behavior of the FPs. That said, we have used the polyQ fusions to show that yFusionRed protein levels are decreased using dot blot (see #3) and this appears to mask its ability to aggregate and consequently generate a toxic phenotype (Figure 4).***

1. Following from the above point, it is difficult to ascertain whether the differences in expression, toxicity and aggregate extents of the various fluorescent protein tagged Htt constructs (Fig 2) arises from the same issues. To us this would be a more likely (and simpler) explanation than what the authors explain as the protein constructs having differences in inherent toxicity (molecule per molecule). Further clarification of this issue is required. 

*** We agree with the reviewer on this point. We have now performed experiments where polyQ fusions are expressed using multicopy plasmids (2µ) were cells can express up to 50 copies of the constructs. Interestingly, under these conditions, 68Q-yFusionred can indeed aggregate and induce toxicity, albeit to lower extant than ymsfGFP-tagged polyQ counterparts. Therefore it is reasonable to hypothesize that lower expression of yFusionRed (maybe due to increased protein turnover or mRNA level) is responsible for decreased toxicity. Further experiments beyond the scope of this research note will be required to explain the relative lower expression of FusionRed in yeast.***

1. The level of reproducibility should be indicated by including at least 3 independent replicates for the dot blots and growth assays (in Fig 2).  Also, the dot blots should be quantified with the appropriate statistical methods.

***We have now added quantification of the dot blots and perfrom quantitative growth assays in liquid media to complement the spot assays on agar plates.***

1.  In Fig 1D, the authors report a significant difference in the median fluorescent intensities using a Student’s t-test to compare only two of the RFPs examined. It is suggested that an ANOVA would be more appropriate because there are more than two groups of interest. Based on the data provided, our ANOVA reports a higher degree of statistical significance for the comparison currently reported (yemRFP v ymScarlet, **) and also supports a significant difference between ymFusionRed and ymScarlet (***).

***We thank the reviewer and have now added appropriate statistical analysis.***

******

 Minor suggestions: 

1. This sentence in the abstract has an unclear meaning as it implies ymScarlet is inherently affected by polyQ: “We found that unlike yemRFP and yFusionRed, the synthetically engineered ymScarlet displayed severe polyQ toxicity and aggregation similar to what is observed for green FP variants.”  Thus the sentence should be revised.

***We have modified accordingly.***

1. Additional details for the Dot blot method (secondary antibody for Pkt-1, dilutions) would enhance reproducibility

***We have added additional information.***

1. Include dimension for scale bar in Figure 2D.

***We have added the information.***
